# Association Between Automated 3D Measurement of Coronary Luminal Narrowing and Risk of Future Myocardial Infarction

**DOI:** 10.1007/s12265-024-10500-2

**Published:** 2024-03-01

**Authors:** Alessandro Candreva, Maurizio Lodi Rizzini, Karol Calò, Mattia Pagnoni, Daniel Munhoz, Claudio Chiastra, Jean-Paul Aben, Stephane Fournier, Olivier Muller, Bernard De Bruyne, Carlos Collet, Diego Gallo, Umberto Morbiducci

**Affiliations:** 1 PolitoBIOMed Lab, Department of Mechanical and Aerospace Engineering, Politecnico Di Torino, Corso Duca Degli Abruzzi 24, 10129 Turin, Italy; 2 Department of Cardiology, Zurich University Hospital, Zurich, Switzerland; 3 Department of Cardiology, Lausanne University Hospital, Lausanne, Switzerland; 4 Cardiovascular Center Aalst, OLV-Clinic, Aalst, Belgium; 5 Pie Medical Imaging, Maastricht, Netherlands

**Keywords:** Quantitative coronary angiography, Coronary artery disease, Myocardial infarction, Translesional pressure gradient, Minimum lumen ratio

## Abstract

This study focuses on identifying anatomical markers with predictive capacity for long-term myocardial infarction (MI) in focal coronary artery disease (CAD). Eighty future culprit lesions (FCL) and 108 non-culprit lesions (NCL) from 80 patients underwent 3D quantitative coronary angiography. The minimum lumen area (MLA), minimum lumen ratio (MLR), and vessel fractional flow reserve (vFFR) were evaluated. MLR was defined as the ratio between MLA and the cross-sectional area at the proximal lesion edge, with lower values indicating more abrupt luminal narrowing. Significant differences were observed between FCL and NCL in MLR (0.41 vs. 0.53, *p* < 0.001). MLR correlated inversely with translesional vFFR (*r* =  − 0.26, *p* = 0.0004) and was the strongest predictor of MI at 5 years (AUC = 0.75). Lesions with MLR < 0.40 had a fourfold increased MI incidence at 5 years. MLR is a robust predictor of future adverse coronary events.

**Graphical Abstract:**

The minimum lumen ratio (MLR) is defined as the ratio between the minimum lumen area (MLA) and the cross-sectional area at the proximal edge of the lesion (PROXA). A lower MLR suggests a more pronounced luminal narrowing upstream of the MLA. This specific anatomical lesion characteristic correlates with higher translesional pressure gradients and has been found to be highly predictive of lesion destabilization over a 5-year period. Notably, lesions exhibiting MLR values below 0.399 were associated with a fourfold increase in the incidence of myocardial infarction (MI) within the same timeframe.

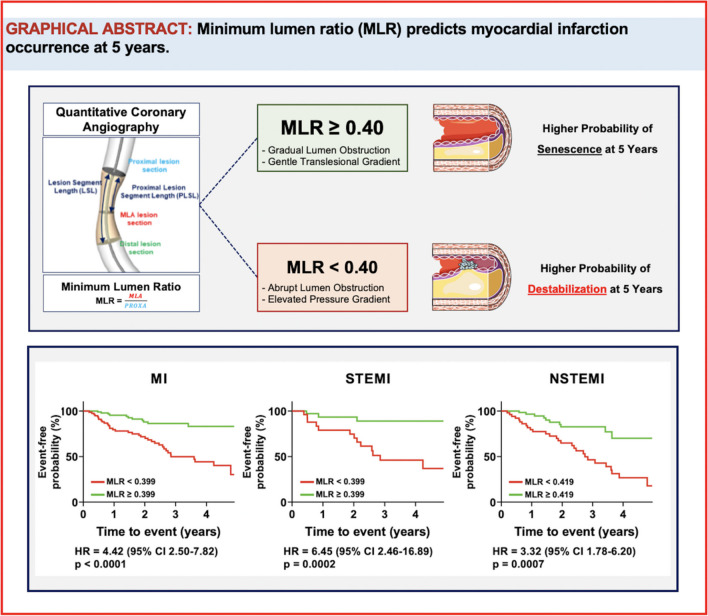

**Supplementary Information:**

The online version contains supplementary material available at 10.1007/s12265-024-10500-2.

## Introduction

The intricate interplay between coronary anatomy and hemodynamics has assumed a central role in the management of coronary artery disease (CAD) [1]. Quantitative coronary angiography (QCA) offers a powerful tool to objectively measure and evaluate anatomical parameters such as lesion length or lumen diameter, cornerstone metrics of clinical decision-making in the diagnosis and treatment of CAD [2]. Nevertheless, the visual assessment conducted by operators often exhibits poor alignment with the functional significance of the lesion [3]. Accordingly, QCA-based computational techniques for the estimation of intracoronary pressure distribution and fractional flow reserve (FFR) have emerged as viable solutions for an objective assessment of lesion hemodynamic relevance without the need for the positioning of pressure wires or the induction of pharmacological hyperemia [4]. More recently, the investigation of intracoronary pressure pullback gradients (either invasively or computationally) has added a new dimension to the evaluation of intracoronary physiology, highlighting how the magnitude and the focality of the pressure drop across the diseased vessel closely relate with high-risk plaque features [5] and adverse events during follow-up [6, 7].

In this context, the present study evaluates anatomical and functional parameters derived from angiographic three-dimensional (3D) QCA and vessel FFR (vFFR) analysis and interrogates their predictive capacity for the occurrence of myocardial infarction (MI) at 5 years.

## Methods

### Study Population

The present investigation was performed on a previously presented retrospective longitudinal multicentric registry [6]. Briefly, patients presenting with an angiographically confirmed acute MI (*index*) and a previous coronary angiography (*baseline*) performed from 1 month to 5 years before the index event were included. Baseline coronary angiograms were visually inspected for vessels presenting mild lesions (≤ 50% diameter stenosis). The lesions on the vessel site of the future MI were labelled as *future culprit lesions* (FCL), whereas the lesions in the other coronary arteries as *non-culprit lesions* (NCL). Vessels were excluded from the analysis if presenting either an aortocoronary bypass (ACBP), a stent, a lesion at the level of a bifurcation or at the ostium, or if there were no identifiable mild lesions at the visual assessment. Additionally, poor angiography quality or the absence of angiographic projections were among the other exclusion criteria considered [6]. Patients with either ST segment elevation MI (STEMI) or non-ST segment elevation MI (NSTEMI) were included. The study protocol conformed to the ethical guidelines of the 1975 Declaration of Helsinki and was approved by the local ethics committee (CER-VD 2019–01932).

### Quantitative Coronary Angiography and Lesion Geometry Characterization

**Fig. 1:**
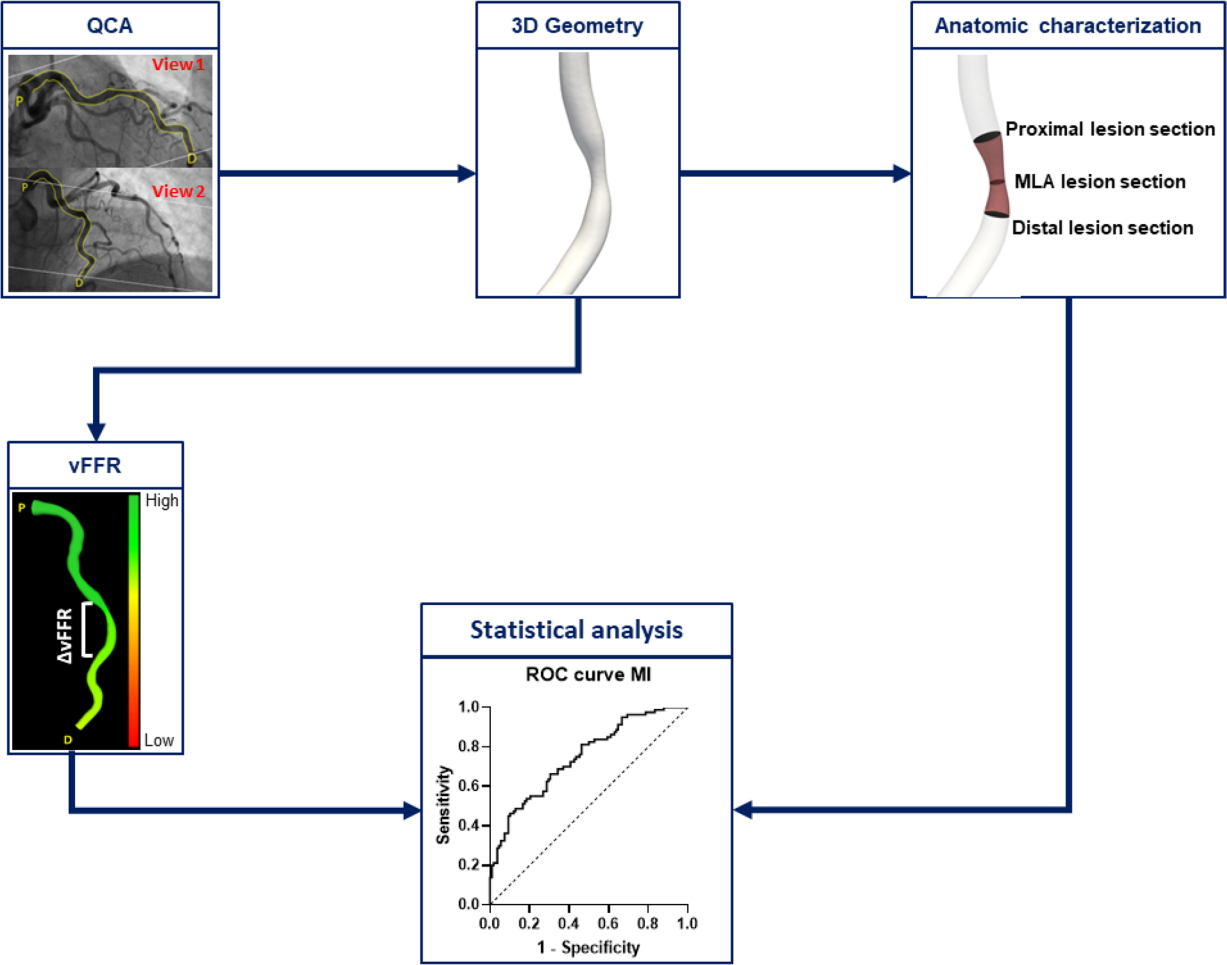
Workflow of the study. Patient-specific three-dimensional vessel reconstructions were obtained from angiographic images and used to derive geometry parameters, such as the novel tested minimum lumen ratio (MLR), and the vessel fractional flow reserve (vFFR). The predictive power for myocardial infarction (MI) of geometry and vFFR quantities was evaluated by means of receiver operating characteristic (ROC) curves

The workflow of the study is reported in Fig. 1. Both anatomical and functional analyses were conducted in a blinded manner regarding the information on the lesion classification as FCL or NCL, utilizing the CAAS Workstation software (Pie Medical Imaging, Maastricht, the Netherlands). The reconstruction method has been previously validated both in vitro and in vivo, by comparing angiography-based reconstructions with those obtained using intravascular ultrasound imaging [8, 9].

**Fig. 2:**
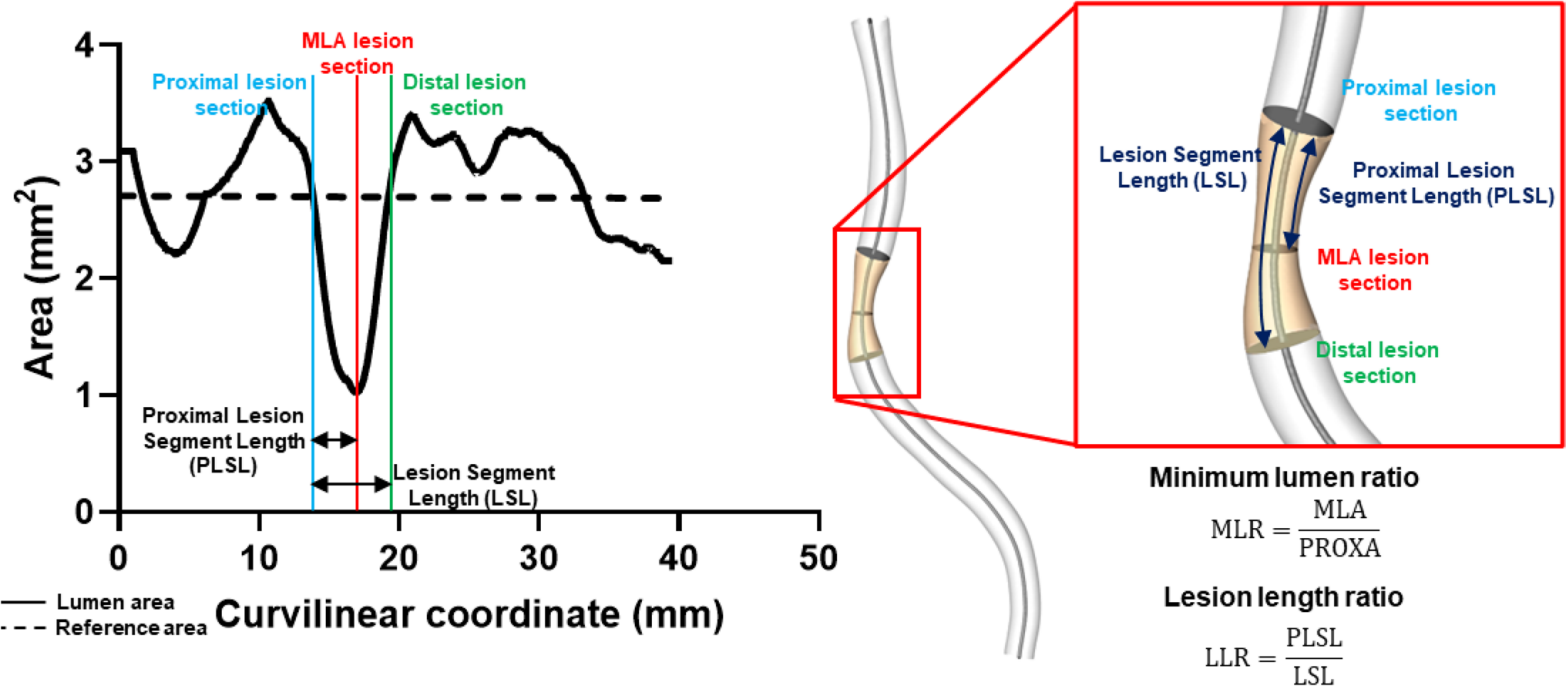
Definition of the minimum lumen ratio (MLR). Proximal and distal lesion sections (or edges) were identified at the intersection between the measured lumen area and reference interpolated area, while the minimum lumen area (MLA) section was identified as the section presenting the lowest surface area value inside the lesion segment length (LSL), defined as the segment between proximal and distal lesion sections. The proximal lesion segment length (PLSL) was defined as the segment between the proximal and MLA sections. The minimum lumen ratio (MLR) was defined as the ratio between the cross-sectional area of the proximal lesion section (PROXA) and the MLA: $\mathrm{MLR}=\frac{\mathrm{MLA}}{\mathrm{PROXA}}$. Finally, the lesion length ratio (LLR) was obtained by the ratio of PLSL and LSL

On each 3D reconstructed vessel geometry, the lesion segment was unambiguously defined as the segment including the minimum lumen area (MLA) and delimited proximally and distally by two edges, identified by the intersection of the QCA area function line with the interpolated reference line (Fig. 2). The proximal lesion segment was defined as the lesion segment upstream of the MLA to the proximal edge of the lesion. The lesion segment length (LSL) and the proximal lesion segment length (PLSL) were defined as the length of the lesion and proximal segments, respectively. The lesion length ratio (LLR) was defined in terms of the ratio between PLSL and LSL.

The surface area of the vessel cross-sections defining the proximal lesion edge (PROXA) and the MLA was used to define the minimum lumen ratio (MLR), as follows (Fig. 2):

1 $${\mathrm{MLR}}\mathrm{=}\frac{\mathrm{MLA}}{{\mathrm{PROXA}}}$$

The MLR is a measure of the narrowing of the proximal segment of the lesion.

### Vessel Fractional Flow Reserve and Translesional Pressure Gradient Quantification

3D QCA-derived vFFR values were obtained along each vessel geometry using the CAAS Workstation vFFR software (Pie Medical Imaging, Maastricht, the Netherlands), whose diagnostic accuracy has been previously validated [10]. The vFFR was evaluated at the distal segment of the vessel, as previously described [10]. The translesional or *delta* vFFR (ΔvFFR) was defined as the difference between the vFFR value at the proximal edge of the lesion and the vFFR value at the distal edge of the lesion.

### Statistical Analysis

Continuous variables are presented as median (interquartile range (IQR)). Mann–Whitney’s U test was used to compare continuous variables between FCL and NCL lesions. For the adjustment for multiple comparisons, Bonferroni’s method was used. The strength of the association of anatomical and functional quantities was evaluated in terms of Spearman’s rank correlation coefficient (*r*). The predictive power of each variable was assessed by receiver operating characteristic (ROC) curves in terms of area under the curve (AUC) and the DeLong test [11]. Youden’s J statistic was applied to infer the best cut-offs from the ROC curves [12], which were then used to dichotomize patients at the time-dependent Kaplan–Meier survival curves. Statistical analyses were performed using SPSS v29 (IBM Corp., Armonk, NY, USA) and R 4.2.1 statistical software (R Foundation for Statistical Computing, Vienna, Austria) assuming a statistical level of significance ≤ 0.05.

## Results

### Patient Population

The study included 188 vessels from 80 patients with a FCL and at least one NCL assessed with both QCA and vFFR (2.35 ± 0.48 vessel/patient). Median time between baseline angiography and MI was 25.9 (21.9–29.8) months. Patients were on average 70.3 years old, mostly men (71.3%), and 65.0% of them experienced a NSTEMI at the time of the index event. Further patient baseline characteristics were reported elsewhere [6] and are summarized in Supplementary Table 1.

### Results of the QCA and vFFR Analysis

The analysis of anatomo-functional parameters yielded notable disparities between the FCL and NCL groups (Table 1). Specifically, the MLR presented lower median values in the FCL group compared to the NCL group (0.41 vs. 0.53, *p* < 0.001), indicating more marked lumen constriction in the former. Atherosclerotic burden, quantified in terms of MLA, was higher in the FCL group (2.39 vs. 2.98 mm^2^, *p* = 0.027). Translesional vFFR values also differed between groups (0.08 vs. 0.05; *p* < 0.001), as well as absolute vFFR values, with the FCL group presenting lower values (0.84 vs. 0.86; *p* = 0.002). On the other hand, the anatomical descriptors of lesion length PLSL, LSL, and LLR did not present any significant difference between FCL and NCL groups (*p* > 0.05 in all cases). Moderate but significant correlations emerged for MLR with translesional vFFR (MLR vs. ∆vFFR: *r* =  − 0.26, *p* < 0.001) and distal vFFR (MLR vs. vFFR: *r* = 0.15, *p* = 0.040) (Supplementary Fig. 1).

**Table 1:** Results of the quantitative coronary angiography (QCA) and the vessel fractional flow reserve (vFFR) analysis (vessel *N* = 188)

	Total	FCL	NCL	Adjusted *p*-value
	Vessel *N* = 188	Vessel *N* = 80	Vessel *N* = 108	
MLR	0.48 (0.40–0.57)	0.41 (0.34–0.51)	0.53 (0.43–0.60)	< 0.001
MLA, mm^2^	2.82 (1.95–3.70)	2.39 (1.82–3.25)	2.98 (2.19–3.98)	0.027
PROXA, mm^2^	5.88 (4.16–7.84)	5.96 (4.12–7.90)	5.56 (4.16–7.78)	0.810
DISTA, mm^2^	5.02 (3.50–6.75)	5.21 (3.54–6.94)	5.01 (3.48–6.52)	0.605
LLR	0.54 (0.36–0.68)	0.53 (0.34–0.68)	0.54 (0.39–0.68)	0.710
PLSL, mm	6.52 (3.50–10.16)	6.54 (3.47–9.99)	6.50 (3.52–11.03)	0.568
LSL, mm	13.97 (9.52–21.10)	14.20 (9.95–20.74)	13.53 (9.51–21.10)	0.986
∆vFFR	0.06 (0.03–0.11)	0.08 (0.04–0.13)	0.05 (0.03–0.08)	< 0.001
vFFR	0.86 (0.81–0.91)	0.84 (0.75–0.90)	0.86 (0.82–0.92)	0.002

Values are represented as median and interquartile range (25th percentile–75th percentile). FCL, future culprit lesion; LLR, lesion length ratio; LSL, lesion segment length; MLR, minimum lumen ratio; NCL, non-culprit lesion; PLSL, proximal lesion segment length; S_MLA_, cross-section of the minimum lumen area; DISTA, cross-sectional area at the distal lesion edge; PROXA, cross-sectional area at the proximal lesion edge; vFFR, vessel fractional flow reserve; ∆vFFR, translesional vFFR difference

### Prediction of Future MI

**Fig. 3:**
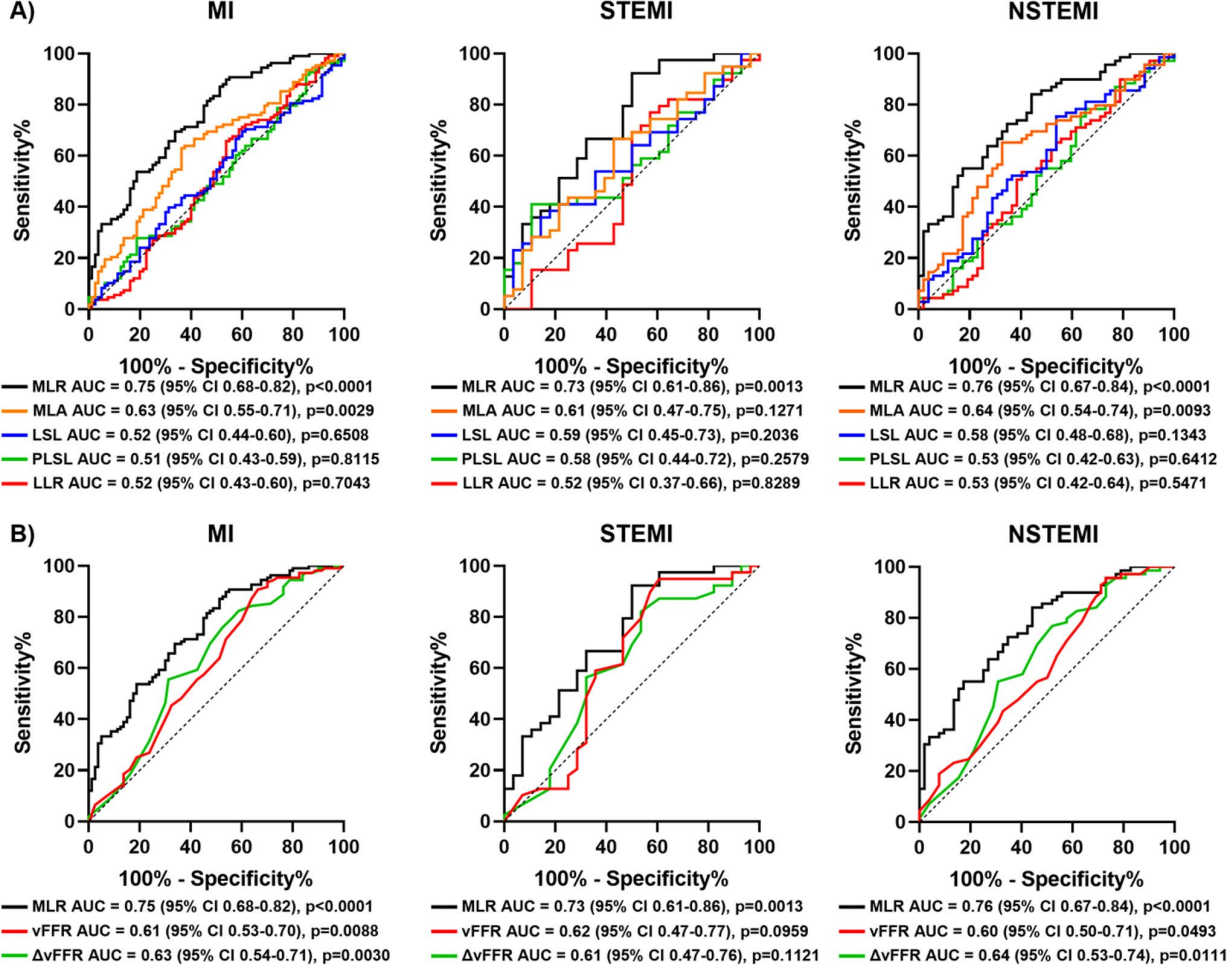
Prediction models for myocardial infarction occurrence at 5 years. Receiver operating characteristic (ROC) curves and estimates of the investigated anatomical (**A**) and functional (**B**) lesion severity descriptors for the prediction of any type of myocardial infarction (MI, left quadrants), ST-segment elevation myocardial infarction (STEMI, central quadrants) or non-ST-segment elevation myocardial infarction (NSTEMI, right quadrants) at 5 years. Abbreviations: ∆vFFR, delta vessel fractional flow reserve; AUC, area under the curve; LLR, lesion length ratio; LSL, lesion segment length; MLA, minimum lumen area; MLR, minimum lumen ratio; PLSL, proximal lesion segment length; vFFR, vessel fractional flow reserve

The luminal surface narrowing in the proximal segment of the lesion, quantified in terms of MLR, emerged as strong predictor for MI (MLR AUC = 0.75, 95% CI 0.68–0.82, *p* < 0.0001), overcoming all the tested anatomical lesion descriptors and in particular the MLA (MLA AUC = 0.63 95% CI 0.55–0.71, *p* = 0.003) (Fig. 3A and Supplementary Fig. 2). The other tested anatomical descriptors of lesion length PLSL, LSL, and LLR did not exhibit significant predictive capacity of future MI (Fig. 3A). Moreover, MLR outperformed also functional lesion severity descriptors (∆vFFR AUC = 0.63, 95% CI 0.54–0.71, *p* = 0.003 and vFFR AUC = 0.61, 95% CI 0.53–0.70, *p* = 0.009) (Fig. 3B). A similar trend was observed also when categorizing the ACS type in STEMI or NSTEMI.

**Fig. 4:**
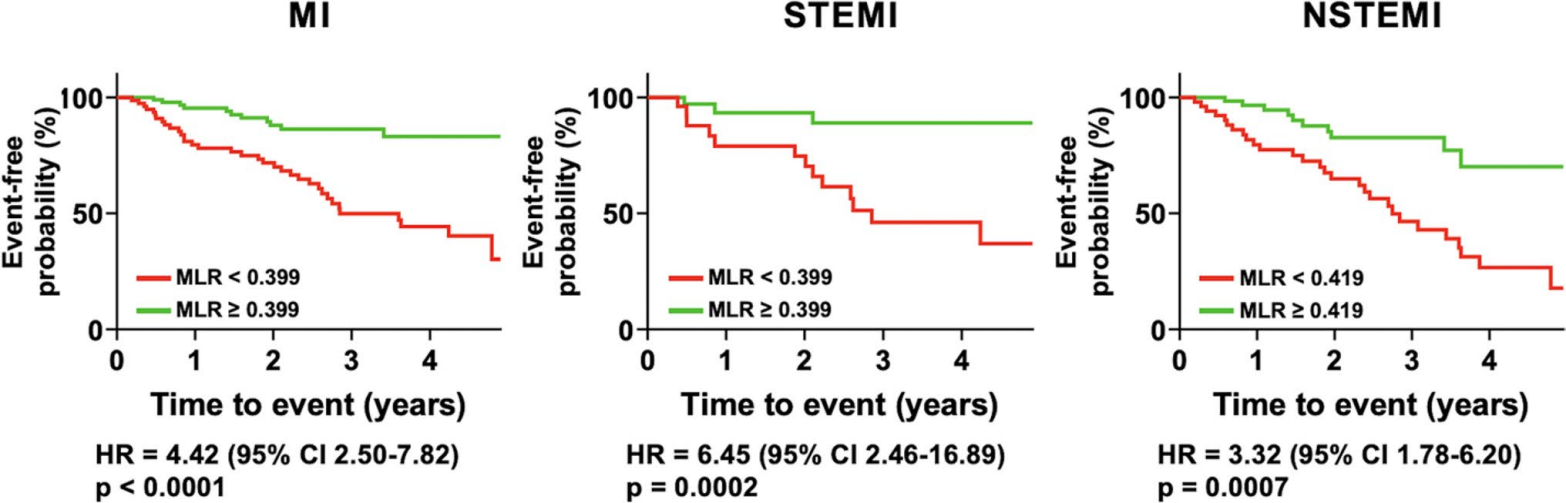
Time-to-event curves. Kaplan–Meier curves and hazard ratios (HR) for low or high minimum lumen ratio (MLR) for the occurrence of any type of myocardial infarction (MI, left quadrant), ST-segment elevation myocardial infarction (STEMI, central quadrant) or non-ST-segment elevation myocardial infarction (NSTEMI, right quadrant) at 5 years

At the survival analysis, lesions exhibiting MLR values below the empirical threshold of 0.399 at the baseline had a fourfold increased incidence of MI at 5 years (hazard ratio (HR) = 4.42, 95% CI 2.50–7.82, *p* < 0.0001) (Fig. 4).

## Discussion

The primary findings of this study underscore the centrality of anatomical parameters derived from angiographic three-dimensional QCA in predicting the occurrence of adverse coronary events at a 5-year mark. This research bridges the gap between angiography and intracoronary physiology, providing new insights into the interplay of anatomical and functional metrics in determining the risk of future MI. In particular, we observed significant differences in the MLR, MLA, ΔvFFR, and vFFR values between mild coronary lesions that will be the site or not of a subsequent acute coronary event. Among these parameters, MLR emerged as the strongest predictor of MI at 5 years. Lesions characterized by lower MLR values, hence presenting with a sharper luminal narrowing, exhibited a substantially increased risk of destabilization.

### Anatomical Lesion Severity Descriptors: Spotlight on Luminal Narrowing

Previous studies suggested that the severity of luminal narrowing at the site of a lesion is critical in the pathogenesis of plaque progression and destabilization and is predictive of adverse events at the follow-up [13, 14]. Area stenosis and MLA—defined either angiographically or by intravascular imaging—emerged as established marks of CAD severity [15, 16]. More recently, plaque components and perivascular tissue elements were clinically linked with lesion vulnerability and adverse events [14, 17, 18].

In the present study, anatomical features of lesions exiting clinical quiescence and provoking overt myocardial events were compared to those of quiescent ones from the same patients, thereby providing a patient-specific control for systemic biological confounders. As a result, the ratio between the cross-sectional areas at the lesion’s proximal edge and at the lesion’s narrowest part, captured by the MLR, provided a robust prediction for future adverse coronary events, outperforming traditional anatomical descriptors of lesion severity. In fact, by mathematically adjusting the narrowest lesion cross-sectional area (i.e., the MLA) with the cross-sectional area of the lesion proximal edge, MLR encompasses the antegrade disease progression along the vessel. In this way, MLR accounts for the narrowing of the diseased vessel segment. Considering previously published data on 5-year predictivity for MI in the same patient population, MLR showed to overcome percental area stenosis (%AS) in performance (%AS AUC = 0.65 (95% CI 0.57–0.73) vs. MLR AUC = 0.75 (95% CI 0.68–0.82)) [6].

Besides that, rather than the disease’s progression along the vessel’s axis, it was the radial inward protrusion of the vessel wall and the subsequent luminal narrowing that correlated with the studied clinical outcomes. In fact, anatomical descriptors of lesion length, including PLSL, LSL, and LLR, did not differ among the FCL and NCL groups and presented no predictive capacity for future MI, while MLR and MLA emerged as significant predictors. The lack of significant variance in PLSL between the FCL and NCL groups particularly highlights the MLR’s independence from lesion length information when assessing myocardial infarction risk. These insights suggest that focusing on the severity of luminal narrowing, rather than the length of the lesion, might be more critical in predicting myocardial events.

Notably, the methods of the present investigation and the MLR computation could be translated to any invasive and non-invasive imaging modality able to produce a 3D vessel reconstruction (including computer tomography angiography, optical coherence tomography, and intravascular ultrasound). The here proposed angiography-based approach might still represent the optimal compromise between the accuracy of vessel reconstructions (lower with non-invasive imaging modalities) and costs (higher in invasive intra-vascular imaging) [19].

### A Novel Anatomo-Functional Lesion Severity Descriptor: The Role of MLR in Coronary Events Prediction

Intracoronary pressure measurement has evolved from a static distal measure (FFR) to a dynamic evaluation along the length of the vessel, thanks to pullback pressure measurements [20]. The ability to functionally identify focal or diffuse CAD phenotypes by analyzing the loss of pressure along the wired vessel has given rise to the concept of local pressure drop patterns. The focal CAD pattern was associated with higher plaque burden, lipid-rich composition, and vulnerability traits [21] and correlated with better angina relief post-percutaneous treatments when compared with the diffuse CAD phenotype [22].

From a functional standpoint, in our study, a pronounced lesion narrowing (identified by a low MLR) was associated with a focal pressure loss pattern along the lesion (evidenced by a high ∆vFFR) as well as with a larger pressure decay along the vessel (denoted by a low vFFR). Moreover, based on the Bernoulli theorem, the lumen narrowing in the converging flow segment of the lesion, quantified by MLR, imparts blood flow energy transformations [23]. The amount of such blood flow energy transformations established within the lesion is expected to impart/exacerbate flow disturbances. These disturbances can be distilled into wall shear stress (WSS) profiles acting at the lesion level, which have been recently identified as strong predictors of lesions culprits of future MI in the here investigated dataset [6, 23].

These considerations suggested that MLR, while deriving from the geometric features of the stenosis, inherently carries a functional significance, which could in turn enhance its clinical predictive potential. Accordingly, in our study, MLR demonstrated superior predictive capacity for the clinical endpoint not only to other anatomical parameters but also to the evaluated functional lesion severity descriptors, such as ΔvFFR and vFFR.

### Study Limitations

This study is not without limitations. It is retrospective in nature, and therefore subject to potential selection bias. The study population is also specific to patients who presented with an acute MI and had prior coronary angiography, which may limit the generalizability of the findings. Furthermore, the potential longitudinal variation and time-dependent effects of cardiovascular risk factors may have influenced our results. However, the inclusion of NCL as an internal control within our study design likely mitigated the impact of these potential confounders. Finally, while our analyses were blinded to the FCL/NCL classification, future prospective studies that incorporate these parameters into the design would further solidify our understanding of these complex relationships.

## Conclusions

Our study findings corroborate recent observations suggesting that the portion of a coronary plaque at the highest risk may not coincide with the MLA [6, 14], underscoring the importance of a meticulous assessment of the entire lesion to provide an accurate risk prediction of subsequent adverse cardiovascular events. MLR served not only as an index of lesion severity but also as a descriptor of its spatial distribution and functional impact, thus linking together anatomical and functional focality, clinically associated with benefits following percutaneous coronary intervention [22]. In this way, a fully automatized methodology based on the long-established QCA solution, with direct translation to any non-invasive and invasive coronary imaging modalities, was tested for the first time in humans and associated to long-term meaningful clinical outcomes. Future research is warranted to validate these findings in larger, prospective cohorts and to confirm the clinical benefit of preemptive treatment strategies targeting lesions with low MLR.

## Supplementary Information

Below is the link to the electronic supplementary material.

Supplementary file1 (DOCX 1060 KB)
