# Supplementary material for: Association Between Automated 3D Measurement of Coronary Luminal Narrowing and Risk of Future Myocardial Infarction
Source: J Cardiovasc Transl Res. 2024 Mar 1;17(4):893–900. doi: 10.1007/s12265-024-10500-2 (PMC11371893; doi:10.1007/s12265-024-10500-2)
Supplement: Supplementary file 1 — Supplementary file1 (DOCX 1060 KB) [file 12265_2024_10500_MOESM1_ESM.docx]

# Supplementary Methods

## Supplementary Table 1

| **Supplementary Table 1. Clinical characteristics (N= 80)** | |
| --- | --- |
| Age, years | 70.3 ±12.7 |
| Female | 23 (28.7%) |
| Type of MI |  |
| - NSTEMI - STEMI | 52 (65.0%)  28 (35.0%) |
| Treatment   - PCI - CABG - Medical | 78 (97.5)  1 (1.3%)  1 (1.3%) |
| Time from baseline ICA, months   - 1 ICA before MI, n - ≥ 2 ICA before MI, n | 25.9 ± 17.7  67 (83.7%)  13 (16.3%) |
| Hypertension | 61 (76.3%) |
| Hyperlipidemia | 63 (78.8%) |
| Diabetes mellitus | 20 (25.0%) |
| Insulin therapy | 7 (8.8%) |
| Smoking | 21(26.3%) |
| LVEF < 55% | 18 (22.5%) |
| eGFR < 60 ml/min/1.73 m^2^ | 18 (22.5%) |
| Prior PCI | 37 (46.3%) |
| Prior stroke | 13 (16.3%) |
| Prior PVD | 18 (22.5%) |
| Aspirin | 61 (76.3%) |
| Statin | 72 (90.0%) |
| P2Y12 inhibitors | 15 (18.8%) |

Clinical characteristics of the studied population at the time of the acute myocardial infarction. Coronary artery bypass graft, CABG; Invasive coronary angiography, ICA; estimated glomerular filtration rate, eGFR; Left ventricle ejection fraction, LVEF; Myocardial infarction, MI; non-ST segment elevation myocardial infarction, NSTEMI; Percutaneous coronary intervention, PCI; Peripheral vascular disease, PVD; ST segment elevation myocardial infarction, STEMI. Table also available elsewhere (*Risk of myocardial infarction based on endothelial shear stress analysis using coronary angiography, Candreva et al. 2022*).

## Supplementary Figure 1. Correlation between vessel tapering and intravascular pressure gradients.


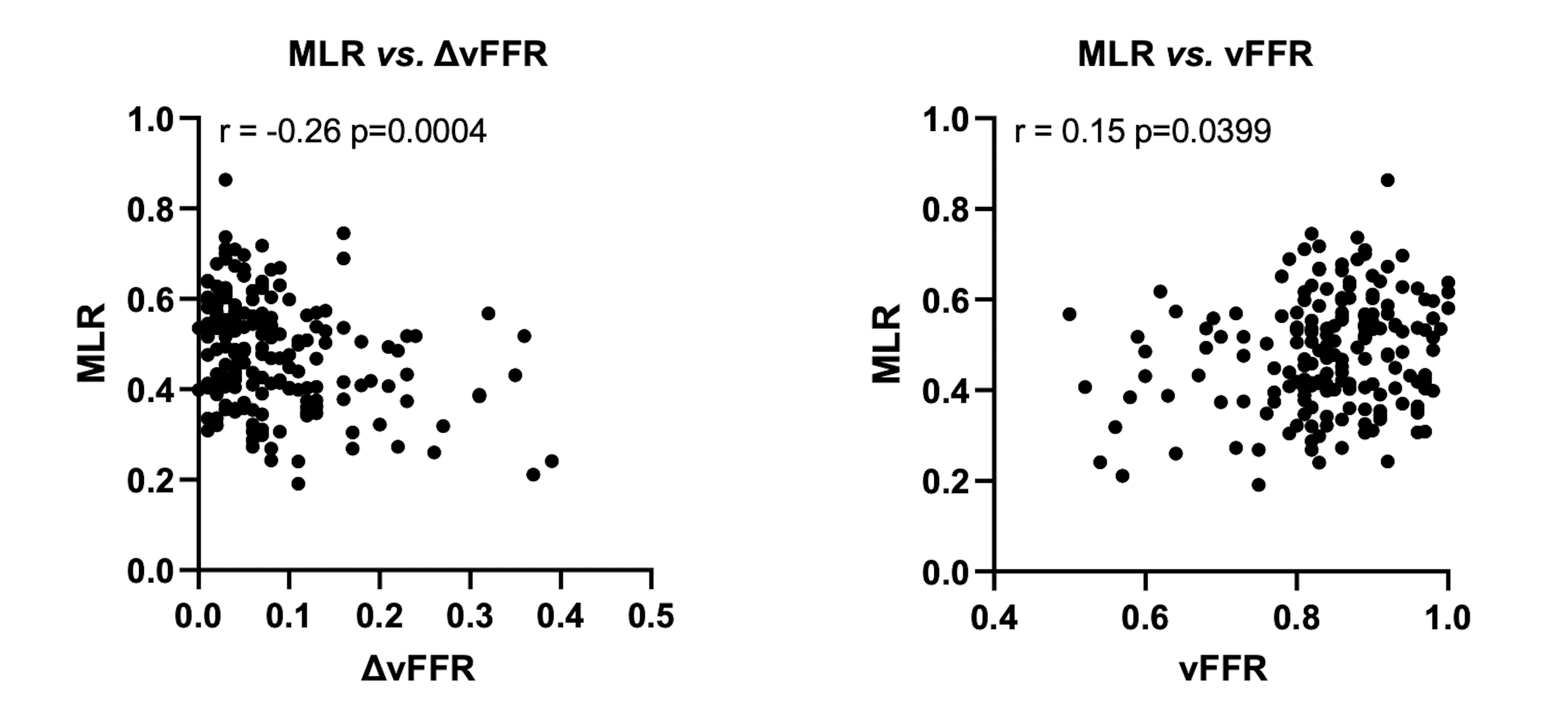


The minimum lumen ratio (MLR) showed significant inverse correlation with the translesional pressure gradient, measured by delta vessel fractional flow reserve (∆vFFR), and significant positive correlation with the distal intracoronary pressure gradient, measured by vessel fractional flow reserve (vFFR).

## Supplementary Figure 2. Results of the ROC curves for the prediction of myocardial infarction for selected anatomical lesion descriptors.


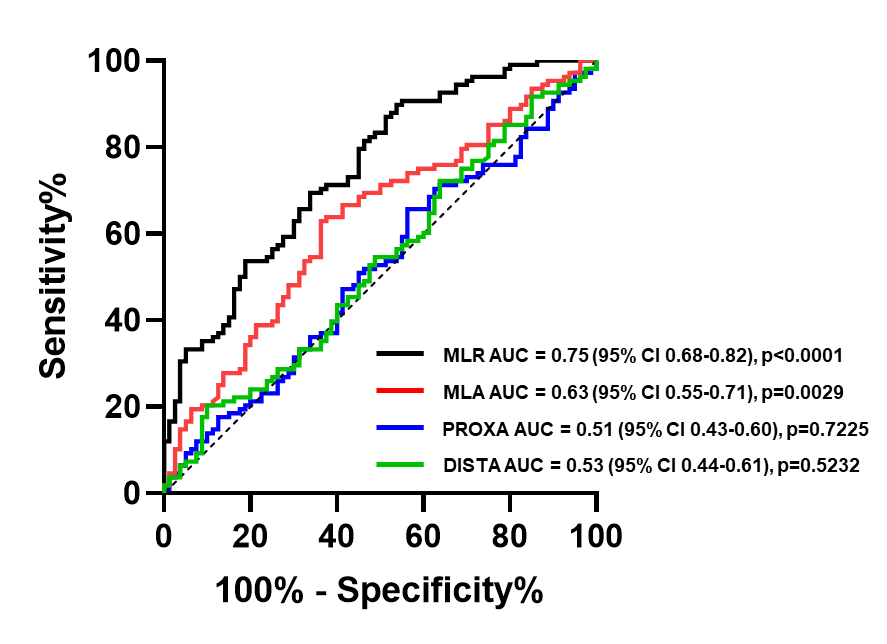


The minimum lumen ratio (MLR) exhibited higher predictive capacity than minimum lumen area (MLA), cross-sectional area at the proximal lesion edge (PROXA) and cross-sectional area at the distal lesion edge (DISTA) for any type of myocardial infarction at 5 years.
